# Skull optical clearing window for *in vivo* imaging of the mouse cortex at synaptic resolution

**DOI:** 10.1038/lsa.2017.153

**Published:** 2018-02-23

**Authors:** Yan-Jie Zhao, Ting-Ting Yu, Chao Zhang, Zhao Li, Qing-Ming Luo, Tong-Hui Xu, Dan Zhu

**Affiliations:** 1 Britton Chance Center for Biomedical Photonics, Wuhan National Laboratory for Optoelectronics-Huazhong University of Science and Technology, Wuhan 430074, China; 2 MoE Key Laboratory for Biomedical Photonics, Collaborative Innovation Center for Biomedical Engineering, School of Engineering Sciences, Huazhong University of Science and Technology, Wuhan 430074, China

**Keywords:** cranial window, dendritic spines, optical clearing, synaptic resolution, two-photon imaging

## Abstract

Imaging cells and microvasculature in the living brain is crucial to understanding an array of neurobiological phenomena. Here, we introduce a skull optical clearing window for imaging cortical structures at synaptic resolution. Combined with two-photon microscopy, this technique allowed us to repeatedly image neurons, microglia and microvasculature of mice. We applied it to study the plasticity of dendritic spines in critical periods and to visualize dendrites and microglia after laser ablation. Given its easy handling and safety, this method holds great promise for application in neuroscience research.

## Introduction

Since the development of transgenic technology^1^, two-photon microscopy has made it possible to image neurons, glia and microvasculature in live mice over time intervals from seconds to years^2, 3, 4, 5, 6, 7, 8^. This technique has become a powerful tool to understand an array of neurobiological phenomena, including the plasticity of individual synapses and neuronal network activity^4, 9^.

However, the strong scattering caused by the skull over the cortex hinders the observation of fluorescently labeled neuronal structures and microvasculature^10, 11^. To overcome this obstacle, various cranial window methods were developed, including the open-skull glass window^12, 13^, the thinned-skull cranial window^14, 15^ and their variants^16, 17, 18, 19, 20, 21, 22, 23, 24, 25, 26, 27, 28^. The open-skull glass window is formed by removing a section of the skull and replacing it with a glass coverslip and may induce an inflammatory response within 3 weeks after surgery^12, 29^. The thinned-skull cranial window is realized by thinning the skull down (to <25 μm). This is a minimally invasive method, but it requires repeatedly thinning the skull due to bone regrowth, which makes it relatively difficult to use^14^. To meet different needs, variants based on craniotomy or skull-thinning surgery have been developed. However, they cannot circumvent the problems inherent to current cranial windows: the associated inflammatory response, the complexities in surgical procedures and high skill requirements for laboratory personnel. Thus, it is urgent to develop a safe and easy-handling cranial window technique for cortical neuroimaging.

The tissue optical clearing technique can reduce the scattering of tissue^30, 31^ and has great potential for solving this problem. By combining it with various optical microscopy techniques, three-dimensional visualization of *ex vivo* brain, spinal cord, etc. with high resolution has been possible, which has helped to achieve major breakthroughs in neuroscience research^32, 33, 34, 35, 36, 37, 38^. In addition, some attempts were made to reduce the scattering of the skull *ex vivo*^36, 37, 39, 40, 41^. However, the optical clearing of the skull *in vivo* has not been sufficiently studied. In recent years, researchers have also performed some *in vivo* experiments to reduce the scattering of the skull, such as applying reagents on the skull to achieve *in vivo* imaging of the cortex^42, 43, 44^. However, it remains impossible to image the cortical structures at synaptic resolution due to the limited optical clearing efficacy of these methods.

Thus, our basic idea is to develop an easy-handling and safe skull optical clearing window (SOCW) for *in vivo* imaging of the cortical structures at synaptic resolution. Combined with two-photon microscopy, this SOCW technique enables us to repeatedly image the dendritic protrusions, microglia processes and blood capillaries in the superficial layers of the cortex. In addition, we investigated the safety of the SOCW technique, concluding that the method is safe. Then, we applied this approach to monitor the plasticity of dendritic protrusions in critical periods and to visualize the changes in dendrites and microglia upon laser injury.

## Materials and methods

### Animals

All animal procedures were approved by the Experimental Animal Management Ordinance of Hubei Province, China and carried out in accordance with the guidelines for humane care of animals. The following mouse strains were used: *Thy1*-YFP-H; *Cx3cr1*^*EGFP/+*^; *Sst-IRES-Cre::*Ai14; and wild-type *C57BL/6*. Transgenic mice were purchased from the Jackson Laboratory (Bar Harbor, ME, USA) and housed and bred in Wuhan National Laboratory for Optoelectronics with a normal cycle (12 h light/dark). Wild-type *C57BL/6* mice were supplied by the Wuhan University Center for Animal Experiment (Wuhan, China). Transgenic mice expressing yellow fluorescent protein under the control of the *Thy1* promoter (*Thy1*-YFP-H) were used for imaging dendritic spines, whereas those expressing enhanced green fluorescent protein in microglia (*Cx3cr1*^*EGFP/+*^) were used for imaging microglia. Transgenic mice expressing red fluorescent protein (*Sst-IRES-Cre::*Ai14) were used for imaging cortical interneurons. Wild-type *C57BL/6* mice were used for imaging the cerebral vasculature labeled with FITC-dextran (50 mg ml^−1^; 2 × 10^6^ molecular weight; Sigma-Aldrich). The *Thy1*-YFP-H mice younger than postnatal day 14 (<P14) have inadequate numbers of brightly labeled cells for *in vivo* imaging. Thus, we selected the mice aged older than P14.

### Reagents

The optical clearing agents (OCAs) used in this study include collagenase (Sigma-Aldrich; T8003), EDTA disodium (Sigma-Aldrich; D2900000) and glycerol (Sigma-Aldrich; 101640026). The OCAs were developed according to the composition of the skull. As we know, the skull consists of an inorganic matrix and an organic matrix. In addition, the main components of the inorganic matrix and organic matrix are calcium hydroxyapatite and collagen, respectively. As the mice grow older, the ratio of inorganic matrix to organic matrix increases. Therefore, for the infantile mice (<P20), collagenase was used to dissolve collagenous fiber; for the elder mice (>P20), EDTA disodium was used to chelate calcium ions (decalcification). In addition, glycerol was used to match the refractive index.

### Schematic diagram of the SOCW technique

**Figure 1:**
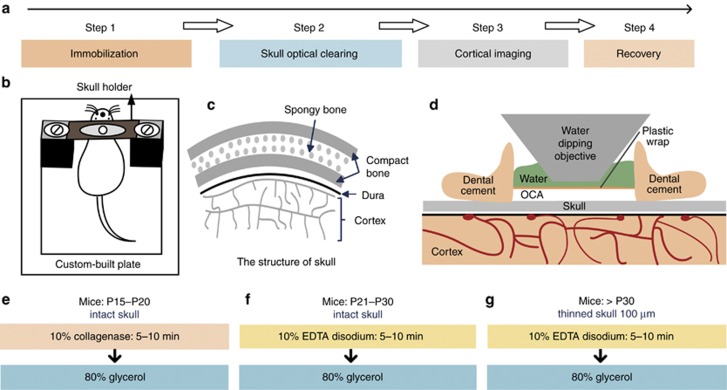
Schematic diagram of SOCW technique for cortical imaging. (**a**) Four steps: immobilization, skull optical clearing, cortical imaging and recovery. (**b**) A custom-built head immobilization device consisting of a skull-holder and a custom-built plate is used to reduce motion artifact during imaging. (**c**) Anatomical structure of mouse skull. (**d**) Schematic of the SOCW. A layer of plastic wrap is placed over the cleared skull to separate the water-immersion objective from the optical clearing agent (OCA). (**e**–**g**) The skull optical clearing methods for mice aged **e** P15–P20, **f** P21–P30, **g** older than P30, which include two steps, the first step is different for mice of different ages. Step 1: for mice aged P15–P20, the intact skull was topically treated with 10% collagenase (w v^−1^) for 5–10 min; for mice aged P21–P30, the intact skull was topically treated with 10% EDTA disodium (w v^−1^) for 5–10 min; for mice older than P30, the thinned skull (100 μm) was treated with 10% EDTA disodium (w v^−1^) for 5–10 min. Step 2: 80% glycerol (v v^−1^) was dropped onto the cleared skull.

Figure 1 shows the schematic diagram of the SOCW technique for cortical imaging. The main experimental procedures include head immobilization, skull optical clearing, cortical imaging and recovery (Figure 1a). After anesthesia, hair removal and scalp incision, mice were fixed with a custom-built immobilization device (Figure 1b) that consisted of a custom-built plate and a skull holder. Further, the skull holder was formed by two conventional double-edged razor blades sandwiching a layer of waterproof paper. On the surface of the skull-holder, dental cement was used to form a reservoir that prevented the OCA from leaking.

The mouse skull is made up of two layers of compact bone sandwiching a layer of spongy bone (Figure 1c), and this structural heterogeneity produces a strong scattering effect. The clearing process takes approximately 15 min and comprises two steps: first, softening the outermost layer of the skull, and then, matching the refractive index. Before imaging, a layer of plastic wrap was placed over the cleared skull to separate the water-immersion objective from the OCA (Figure 1d). After imaging, we gently detached the holder from the skull, thoroughly cleaned the skull and the skin to remove the remaining glue, and sutured the scalp with sterile surgical sutures.

### Skull optical clearing methods

Since components of the skull change with age, various clearing methods were developed for mice of different ages (Figure 1e–1g).

Step 1: For mice aged P15–P20, the intact skull was topically treated with 10% collagenase for 5–10 min (Figure 1e); for mice aged P21–P30, the reagent was replaced by 10% EDTA disodium (Figure 1f). The thickness of the skull increases as mice age; thus, for mice older than P30, we had to thin the skull to approximately 100 μm before clearing and then treat it with 10% EDTA disodium for 5–10 min (Figure 1g).

Step 2: We removed the first reagent above the skull by using a clean cotton ball, and then, 80% glycerol was dropped onto the skull.

### *In vivo* two-photon imaging

*In vivo* images of YFP-expressing dendritic spines/EGFP-expressing microglia/FITC-dextran-labeled cerebral vasculature were acquired by a two-photon microscope (FV1200; Olympus, Tokyo, Japan) with a Mai Tai Ti:sapphire laser (Spectra Physics, Santa Clara, CA, USA) at 925 nm. The output optical power was <40 mW to avoid phototoxicity. Image stacks were obtained with a step size of 1 μm using a water-immersion objective (25 ×, numerical aperture=1.05, working distance=2 mm; Olympus). To relocate the same location, low-resolution images of interest were obtained with a step size of 2 μm. After the initial fluorescence image was acquired, the OCAs were topically applied to the skull, and then, the same area was relocated and reimaged based on the brain vasculature map to assess the optical clearing efficacy.

### Labeling of microglia and astrocytes in mouse cortex

Mice were perfused intracardially with 4% paraformaldehyde. The mouse brains were removed, postfixed and sectioned into 50 μm slices. To examine microglia in the mouse cortex, we used *Cx3cr1*^*EGFP/+*^ transgenic mice, and labeling of astrocytes was performed with antibodies against Glial fibrillary acidic protein (GFAP). Brain sections were stained for GFAP (Proteintech Group, Rosemont, IL, USA; 16825-1-AP; 1:100) using a normal immunostaining protocol. The images were acquired by a Zeiss 710 LSM confocal microscope (Oberkochen, Germany) and a Nikon Eclipse Ni-E wide-field microscope (Tokyo, Japan).

### Laser ablation inside the cortex

The laser ablation inside the cortex was performed by focusing a laser beam on the cortex through the skull. The laser wavelength was 780 nm, and its power was approximately 60–80 mW at the sample. The beam remained at the position of interest for approximately 60 s to create a tiny injury site.

### Data quantification

All data were analyzed by using Image J software that was developed by National Institutes of Health (Bethesda, MD, USA). For spine dynamics in Figure 6, all analyses were performed manually on the raw image stacks. The newly formed spine was the one that was not in the first image but appeared in the second image. The eliminated spine was the one that appeared in the initial image but not in the second image. The elimination and formation rates of spine were, respectively, the number of spines that disappeared or appeared between two imaging time points, relative to the total number of spines in the initial image. In addition, we selected the two-dimensional projections that contained in-focus dendritic segments (with high image quality) to make figures. The quantified data were presented as the mean±s.e.m.

### Data availability

The data that support the findings of this study are available from the corresponding authors upon reasonable request.

## Results and discussion

### Imaging the dendritic spines at synaptic resolution through the SOCW

**Figure 2:**
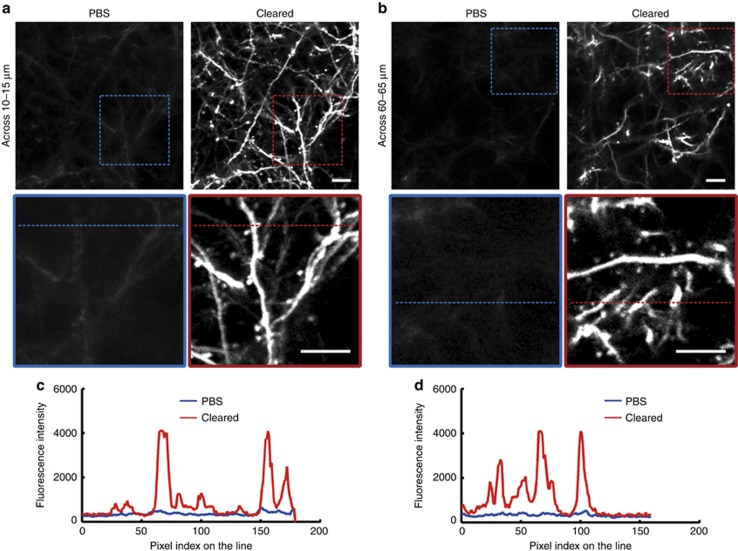
Representative fluorescence images of the dendrites to assess the optical clearing efficacy achieved by the SOCW method. (**a** and **b**) Maximum projections across images **a** 10–15 μm and **b** 60–65 μm below the surface through the intact skull, before and after skull optical clearing (P30, *n*=10 mice). (**c** and **d**) Transverse plots corresponding to the same dendrites and spines indicated by the dashed line in **a** and **b**, showing that the fluorescence intensity is significantly improved. Scale bar=10 μm.

First, we performed *in vivo* experiments to assess the optical clearing efficacy achieved by the SOCW method. We found that the image quality was considerably improved and that it was sufficient for imaging the dendritic spines through the cleared skull (Figure 2 and Supplementary Fig. 1). Figure 2a and 2b demonstrate the typical dendritic spines of *Thy1*-YFP neurons at the depths of 10–15 and 60–65 μm below the pial surface. Figure 2c and 2d show the fluorescence intensity for the same locations indicated by dashed lines in Figure 2a and 2b; we can observe that the image contrast is significantly improved through the cleared skull. Thus, we can achieve imaging of the structures at synaptic resolution through the SOCW.

### The imaging depth through the SOCW

**Figure 3:**
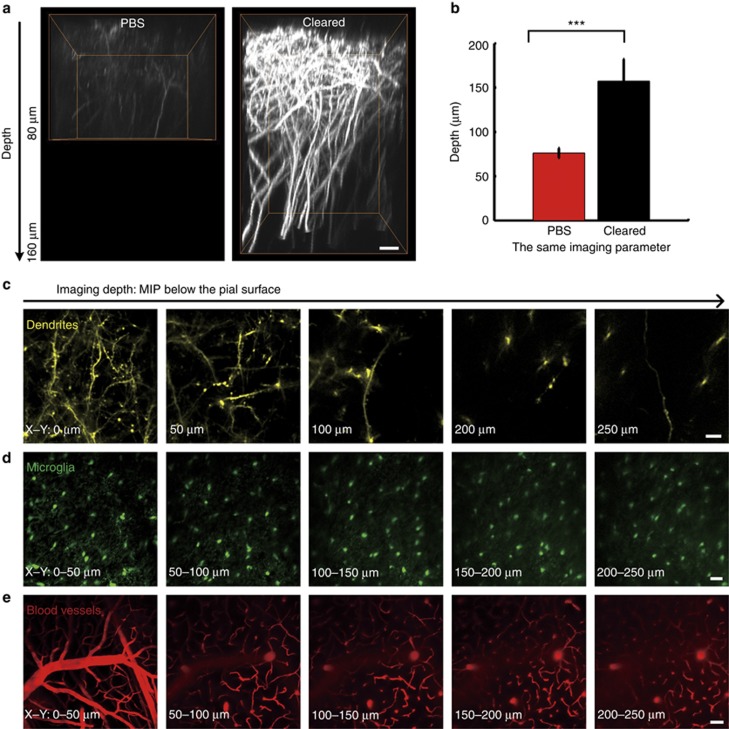
Imaging depth through the SOCW. (**a**) Orthogonal (*x*–*z*) projections of dendrites through the intact skull, before and after skull optical clearing, demonstrating that the depth is obviously enhanced after clearing (the imaging parameter and data processing were the same). Scale bar=10 μm. (**b**) The depth when imaging the dendrites of *Thy1*-YFP neurons, before and after skull optical clearing (P30, *n*=10 mice; statistical method: one-way analysis of variance (ANOVA); *P*<0.001). (**c**–**e**) Imaging depth through the SOCW after optimizing the imaging parameters (P30, *n*=6 mice). (**c**) Dendrites and spines of *Thy1*-YFP neurons at different depths through the SOCW. Scale bar=10 μm. (**d**) Maximum *z*-axis projections across 50 μm of microglia through the SOCW. Scale bar=25 μm. (**e**) Maximum *z*-axis projections across 50 μm of FITC-dextran-filled cerebral vasculature through the SOCW. Scale bar=50 μm.

Then, we evaluated the imaging depth through the SOCW. Figure 3a demonstrates the image along the depth direction (*x*–*z*). In addition to significant increases in fluorescence intensity, the imaging depth also obviously enhanced after clearing. For further quantitative comparison, 10 groups were selected to conduct a statistical analysis, and the mean depth increased approximately twofold (Figure 3b). Considering that confocal microscopy is more common than two-photon microscopy, we also used confocal microscopy to perform the same experiment. Supplementary Fig. 2 shows that the SOCW is also compatible with confocal imaging and that the imaging depth increases from 20 to 60 μm. These images were obtained with consistent parameters to ensure the fair comparison of the results before and after clearing. Furthermore, we could obtain good visibility of dendrites (Figure 3c), microglia (Figure 3d) and blood vessels (Figure 3e) up to 250 μm below the pial surface by optimizing the imaging parameters, which demonstrates that the imaging depth certainly reaches up to 250 μm.

The above results also show that the SOCW technique is compatible with various fluorescent proteins and dyes, including GFP (Figure 3d), YFP (Figure 3c), RFP (Supplementary Fig. 3), FITC (Figure 3e) and tetramethylrhodamine (Supplementary Fig. 4). Therefore, the SOCW technique demonstrates flexibility and widespread applicability.

### The repeatability of the SOCW technique

**Figure 4:**
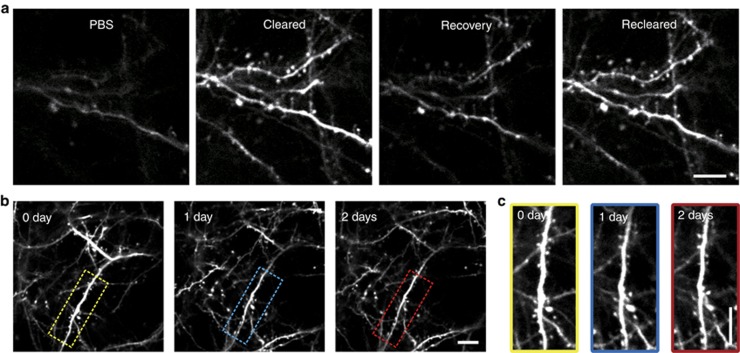
The repeatability of the SOCW. (**a**) Repeated imaging of the dendrites and spines of *Thy1*-YFP neurons within a few hours, which shows that the SOCW is switchable (P30, *n*=10 mice). (**b** and **c**) Repeated imaging of the dendrites **b** and spines **c** of *Thy1*-YFP neurons obtained over a 1-day interval (P28–P30, *n*=10 mice). Scale bar=10 μm.

We also assessed the repeatability of the SOCW technique. The skull could return to the opaque state very quickly after treatment with PBS (phosphate-buffered saline), and retreatment with OCAs made the skull re-clear very quickly (Figure 4a), which shows that the SOCW is switchable. Obviously, bone re-grows with time; if the imaging interval was beyond one day, the time for clearing was slightly longer than the first time. Despite this caveat, we could repeatedly observe the dendrites (Figure 4b), spines (Figure 4c), microglia (Supplementary Fig. 5a) and microvasculature (Supplementary Fig. 5b) through the SOCW. Thus, we could achieve short- and long-term repetitive imaging of the cortex through the SOCW.

### Safety assessment of the SOCW technique

**Figure 5:**
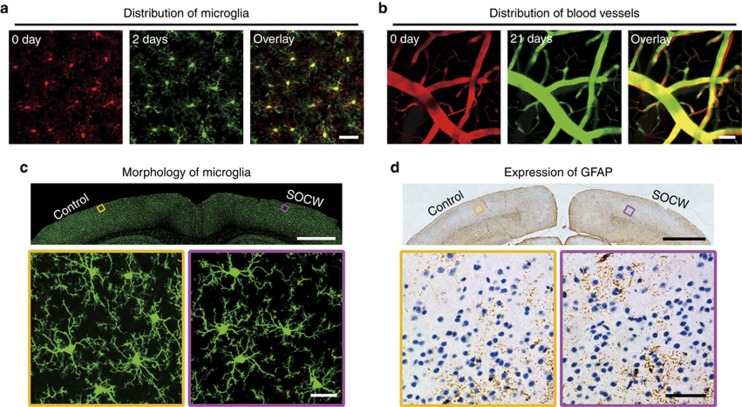
Safety assessment of the SOCW technique. (**a**) Distribution of microglia through the SOCW. After the SOCW technique, microglia remain at the same position (P28, *n*=4 mice). Scale bar=25 μm. (**b**) Maximum projections (*z*-axis) across 10–40 μm of FITC-dextran-filled cerebral vasculature under the SOCW obtained 0 (P28, *n*=4 mice) and 21 days after the treatment. We observe that the cerebrovascular morphology remains nearly unchanged 21 days after forming the SOCW. Scale bar=25 μm. (**c**) Histological images of microglia in the case of the SOCW technique; microglia in both the treated and control sides appear normal (P30, *n*=3 mice). Scale bar=1 mm (above) and 25 μm (below). (**d**) GFAP expression under the SOCW technique. The treated and control hemispheres show similar levels of GFAP expression (P38, *n*=3 mice). Scale bar=1 mm (above) and 50 μm (below).

The above repeated imaging of the same position shows an absence of inflammation. In addition, we performed both *in vivo* (Figure 5a and 5b) and *ex vivo* (Figure 5c, 5d and Supplementary Fig. 6) experiments to assess the safety of this method. First, we observed microglia *in vivo* 0 and 2 days after craniotomy and after clearing, respectively. We found that microglia remained at the same position (Figure 5a) when viewed through the SOCW, which indicates that the SOCW technique does not affect the microenvironment. In contrast, the distribution of microglia seen through the open-skull glass window was completely different (Supplementary Fig. 7a). The craniotomy evidently induced the movement of microglia.

Previous studies showed that the injury of the cortex could trigger outgrowth and remodeling of the surface pial vasculature^19, 45^, so the structures of superficial cerebral vessels under craniotomy showed alterations in topology that may be due to angiogenesis in the meninges caused by inevitable damage. In this work, we also imaged the FITC-dextran-filled cerebral vasculature of *C57BL/6* mice 0 and 21 days after clearing and found that the distribution of cerebral vasculature was nearly the same (Figure 5b). This result indicates that the optical clearing treatment produces no cortical injury.

In general, the microglia are active maximally 2 days after craniotomy^12, 19, 29^, and the expression of GFAP may be upregulated in activated astrocytes 7–14 days after injury^19, 29, 45^. Thus, we carried out *ex vivo* experiments to observe microglia 2 days after surgery and visualize the expression of the GFAP in astrocytes 10 days after surgery. We found that the microglia in both the SOCW and the control hemisphere remained in a non-active state with a highly branched morphology (Figure 5c). Moreover, the density of microglia in cortical layer I/II under the SOCW is consistent with that under the contralateral side (0.99±0.04), which is similar with that through the thinned-skull cranial window^24, 29^. In addition, the GFAP immunostaining patterns exhibit similar levels of GFAP expression for both sides (Figure 5d), which means that the astrocytes were not activated.

In contrast, through the open-skull glass window, the microglia in the superficial layers (Supplementary Fig. 7b) had few processes and extended their branches to the pial surface, similar to the macrophages. This indicates that the microglia had become active^12, 19, 29^. In addition, the density of microglia under the open-skull glass window (cortical layer I/II) was much higher than that under the control sides (1.62±0.25)^24, 29^. In addition, the GFAP immunostaining patterns in the surgery side are very strong and obviously different from those seen in the contralateral hemisphere (Supplementary Fig. 7c).

The above results show that the SOCW method does not induce obvious inflammatory responses in the brain cortex; hence, we can conclude that this SOCW technique is safe and reliable.

### Monitoring the plasticity of dendritic protrusions based on the SOCW

**Figure 6:**
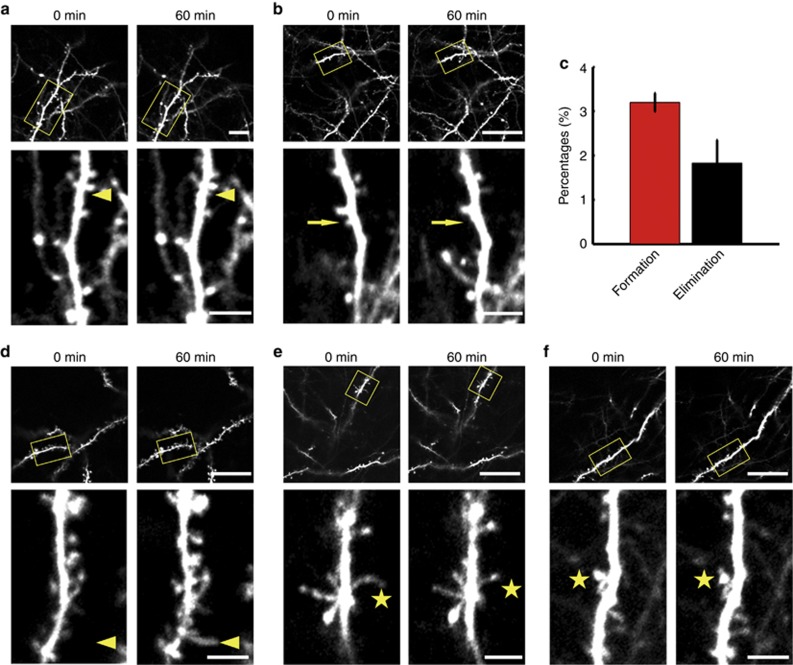
Dynamical monitoring of the plasticity of dendritic protrusions in infantile mice (P19) through the SOCW. Time-lapse images of dendritic branches over an hour (60 min). (**a** and **b**) Dendritic spines can **a** appear and **b** disappear within an hour. (**c**) Percentage of spines formed and eliminated within an hour, according to the SOCW technique (*n*=6 mice). (**d** and **e**) Filopodia can also **d** appear and **e** convert into a spine-like protrusion within an hour. (**f**) The morphology of dendritic spines can change within an hour. The triangle, arrow and asterisk show appearance, disappearance and morphological changes, respectively. Scale bar=25 μm (above) and 5 μm (below).

We next applied this method to dynamically monitor the plasticity of dendritic protrusions in critical periods. To monitor spine turnover, we obtained high-resolution image stacks of the dendritic tuft. The dendritic branches were studded with numerous spines (56.6±1.4 spine per 100 μm), including mushroom-type, stubby and thin spines, as well as long filopodia-like protrusions. By time-lapse imaging dendritic protrusions over an hour (1 h), we found that they exhibited strong motility. Moreover, dendritic spines can suffer changes within 1 h, including appearance (Figure 6a) and disappearance (Figure 6b) of spines and changes of shape (Figure 6f), which points to changes in the wiring of neuronal circuits. We additionally studied the turnover of dendritic spines in the barrel cortex *in vivo* within 1 h after forming the SOCW. We found that spine formation and elimination were 3.20±0.21% and 1.83±0.52%, respectively (Figure 6c, 987 spines, *n*=6 mice). Compared with dendritic spines, filopodia exhibited higher motility. In addition, filopodia can even convert into spine-like protrusions (Figure 6e), demonstrating that filopodia are likely to be the precursors of dendritic spines^46, 47^. These results demonstrate the dynamic nature of these protrusions, which indicates that the plasticity of dendritic protrusions during the third week is very intense.

### Monitoring dendrites and microglia after laser ablation under the SOCW

**Figure 7:**
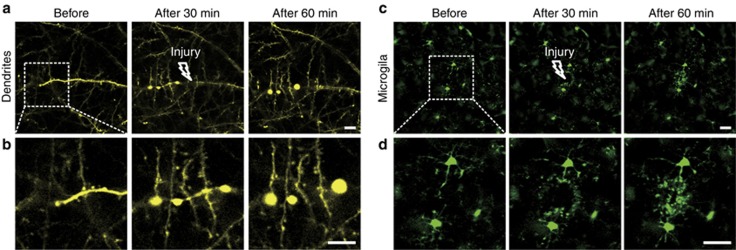
Changes induced by laser ablation in dendrites and microglia. (**a**) Morphology of dendrites after laser injury, obtained using the SOCW technique (P30, *n*=4 mice). After creating a localized ablation inside the cortex with a two-photon laser, nearby dendrites form bead-like structures. (**b**) Magnified images corresponding to the rectangle areas shown in **a**. (**c**) Morphology of microglia after laser injury, obtained using the SOCW method (P30, *n*=4 mice). After creating a localized injury, nearby microglial processes respond immediately with bulbous termini. (**d**) Magnified images corresponding to the rectangular areas shown in **c**. The arrows show the brain injury locations. Scale bar=25 μm.

Laser-induced injury is a widely used injury model because the extent and site of the injury are easily controlled^48, 49^. We also applied this method to monitor dendrites and microglia after laser injury. We found that the dendrites on the laser injury side formed bead-like structures, while the sites that did not suffer damage remained in a normal state (Figure 7a and 7b). Microglia soma did not show any significant movement, but the processes with bulbous termini immediately moved toward the site of injury (Figure 7c and 7d). The SOCW technique enables us to characterize the effects of laser injury on cortical structures.

## Conclusions

In this study, we introduce an easy-handling, safe and effective SOCW for imaging cortical structures at synaptic resolution. Combined with two-photon microscopy, this SOCW technique allowed us to repeatedly visualize the neurons, microglia and microvasculature in the superficial cortical layers.

Compared with the current cranial windows, the SOCW has several advantages. First, the SOCW is easier to handle because the SOCW is established by topically applying reagents on the skull without much thinning or removing of the skull; thus, it may attract more researchers to opt for the SOCW technique. Second, this approach proved to be safe with little risk of producing inadvertent damage or inflammation. Because of its lack of brain inflammation, this technique is also more suitable for the study of the immune cells (microglia) that are highly sensitive to the microenvironment. However, there is a technical and practical limitation of the SOCW. The imaging depth is limited to the first 250 μm below the pial surface due to the existence of the skull, which is not comparable with traditional cranial windows. Therefore, the SOCW with no need for much thinning or removal of the skull, as an alternative technique, could permit us to image cortical cells and vasculature under extremely similar environments with the normal state of the brain.
