# Author Correction: Skull optical clearing window for in vivo imaging of the mouse cortex at synaptic resolution

**DOI:** 10.1038/s41377-018-0021-1

**Published:** 2018-05-18

**Authors:** Yan-Jie Zhao, Ting-Ting Yu, Chao Zhang, Zhao Li, Qing-Ming Luo, Tong-Hui Xu, Dan Zhu

**Affiliations:** 1 Britton Chance Center for Biomedical Photonics, Wuhan National Laboratory for Optoelectronics-Huazhong University of Science and Technology, Wuhan, Hubei 430074 China; 2 MoE Key Laboratory for Biomedical Photonics, Collaborative Innovation Center for Biomedical Engineering, School of Engineering Sciences, Huazhong University of Science and Technology, Wuhan, Hubei 430074 China

**Correction to**: *Light: Science & Applications* (2018) **7**, 17153; 10.1038/lsa.2017.153; Article published online, 23 February 2018.

In the version of this article originally published, in the “Materials and methods” section under subheading “Reagents”, there are errors about the information of reagents. And the original and corrected versions are shown below.

Materials and methods, Page 2

“The optical clearing agents (OCAs) used in this study include collagenase (Sigma-Aldrich; T8003), EDTA disodium (Sigma-Aldrich; D2900000), and glycerol (Sigma-Aldrich; 101640026).”

Should read

“The optical clearing agents (OCAs) used in this study include collagenase (Sigma-Aldrich; C0130), EDTA disodium salt (Sigma-Aldrich; D2900000) and glycerol (Sigma-Aldrich; G5516).”

We apologize for any inconvenience this may have caused.

