# Supplementary material for: Skull optical clearing window for in vivo imaging of the mouse cortex at synaptic resolution
Source: Light Sci Appl. 2018 Feb 23;7:17153–. doi: 10.1038/lsa.2017.153 (PMC6060065; doi:10.1038/lsa.2017.153)
Supplement: Supplementary Information [file lsa2017153x1.pdf]

SUPPLEMENTARY INFORMATION for

Skull optical clearing window for *in vivo* imaging of the mouse cortex at synaptic resolution

Yanjie Zhao[1,2], Tingting Yu[1,2], Chao Zhang[1,2], Zhao Li[1,2], Qingming Luo[1,2], Tonghui Xu[1,2*], Dan Zhu[1,2*]

[1] Britton Chance Center for Biomedical Photonics, Wuhan National Laboratory for Optoelectronics-Huazhong University of Science and Technology, Wuhan, Hubei 430074, China

[2] MoE Key Laboratory for Biomedical Photonics, Collaborative Innovation Center for Biomedical Engineering, School of Engineering Sciences, Huazhong University of Science and Technology, Wuhan, Hubei 430074, China

*D Zhu, Email: dawnzh@mail.hust.edu.cn; TH Xu, Email: xutonghui@hust.edu.cn, Tel: +86 027 87792033

Imaging the dendritic spines in infantile and adult mice through the SOCW

We keep the skull of infantile mice intact without thinning (**Supplementary Fig. 1a**). The composition and thickness of the skull changes with age, which lead to strong scattering and further reduce the two-photon imaging performance. To visualize the dendritic spines of mice older than P30, we have to thin the skull to about 100 μm before clearing (**Supplementary Fig. 1b**). **Supplementary Fig. 1c–d** shows that the maximum projections of *Thy1*-YFP neurons across 10–15 μm images below the surface through the skull of mice aged P19 (**Supplementary Fig. 1c**) and P60 (**Supplementary Fig. 1d**) before and after skull optical clearing. We can see that the image quality is obviously improved and it is sufficient for imaging dendritic spines after treatment with OCAs.

Using one-photon to illuminate

We used one-photon microscopy to image the dendrites of *Thy1*-YFP neurons (P30) through the intact skull. After clearing, the image quality is obviously improved and the imaging depth increases from 20 μm to about 60 μm (**Supplementary Fig. 2**). Therefore, this method is also effective for confocal microscopy. The images were acquired by Zeiss 780 LSM confocal microscope with a water-immersion objective (20×, numerical aperture = 1.0, working distance = 1.8 mm, Zeiss). And the 488 nm laser was used to illuminate.

Imaging the interneurons expressing RFP with the SOCW

We used transgenic mice *Sst-IRES-Cre::*Ai14 to image the cortical interneurons expressing red fluorescent proteins (RFP). Before clearing, we could hardly get any information about interneurons, but after clearing, the interneurons across 100–200 μm below the surface could be obtained. **Supplementary Fig. 3** shows the images of mice aged P30. Thus, this method is also compatible for RFP as well.

Dual-color imaging

Dual-color imaging could also be realized under SOCW (**Supplementary Fig. 4**). The blood vessels of transgenic mice were labeled with fluorescence dye (Tetramethylrhodamine-conjugated dextran) via tail vein injection. **Supplementary Fig. 4a–b** shows that the cerebral vasculature can be imaged at the same time as

dendrites (**Supplementary Fig. 4a**) and microglia (**Supplementary Fig. 4b**).

Repeated imaging of microglia and brain micro-vessels

And we also repeatedly imaged microglia in mice *Cx3cr1*$^{EGFP/+}$ (P28) and subsurface brain micro-vessels in *C57BL/6* mice (P28) in which the plasma had been stained with fluorescein-conjugated dextran. **Supplementary Fig. 5a–b** shows representative fluorescence images of GFP-expressing microglia cells, FITC-dextran labeled cerebrovascular in the cortex 0 d, 2 d and 21 d after clearing. Thus the clearing technique enables us to repeatedly image microglia and micro-vessels.

Safety assessment of the SOCW in adult mice

The microglia and astrocytes through the SOCW in adult mice remain in non-active state. We found that the microglia in both the SOCW and the control hemisphere remained in non-active state with a highly branched morphology (**Supplementary Fig. 6a–b**). **Supplementary Fig. 6c** shows that the GFAP immunostaining patterns exhibit similar levels of GFAP expression for the both sides, which means the astrocytes are not activated. It means that the optical clearing does not induce an inflammation response in adult mice.

Safety assessment of the craniotomy technique

First, the distribution of microglia 0 and 2 d after craniotomy was completely different (**Supplementary Fig. 7a**). Second, the morphology of microglia (**Supplementary Fig. 7b**) and the expression of the glial fibrillary acidic protein in astrocytes (**Supplementary Fig. 7c**) obviously changed. The results show that the craniotomy may induce inflammatory responses.

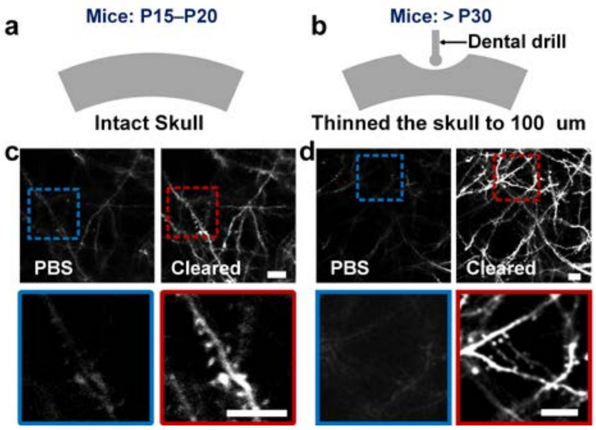

Supplementary Figure 1 Imaging the dendritic spines of *Thy1*-YFP neurons with the SOCW in infantile mice (P19) and adult mice (P60). (**a**) We keep the skull (infantile mice) intact without thinning. (**b**) The dental drill is used to thin the mouse skull (>P30) to about 100 μm. (**c–d**) Maximum projections of *Thy1*-YFP neurons of mice aged P19 (**c**) and P60 (**d**) across 10–15 μm images below the surface through the intact skull and 100 μm thinned skull, before and after skull optical clearing. Scale bar, 10 μm.

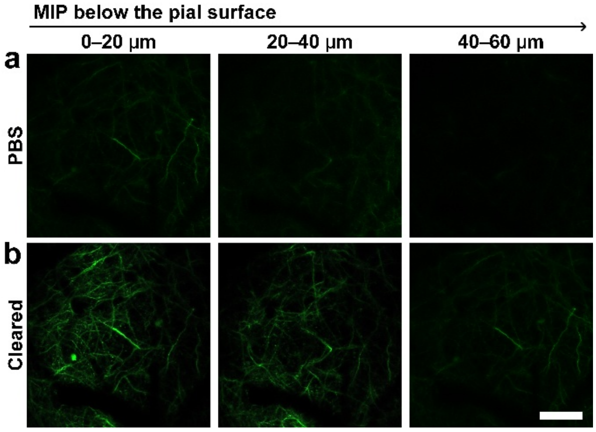

Supplementary Figure 2 Imaging the dendrites of *Thy1* YFP neurons (P30) by using one-photon microscopy through the intact skull, before and after skull optical clearing. (**a–b**) Maximal projections of dendrites at different depths, the images were obtained with consistent parameters to ensure the fair comparison of the results before and after clearing. Scale bar, 100 μm.

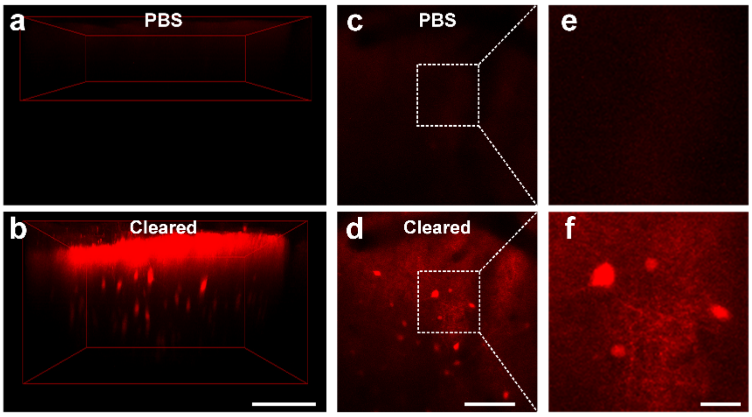

Supplementary Figure 3 The cortical interneurons expressing red fluorescent proteins in mice aged P30 can also be obtained with the SOCW technique. (**a–b**) Orthogonal (x-z) projections of interneurons through the intact skull, before and after skull optical clearing, demonstrating that the imaging depth enhances obviously after clearing. (**c–f**) Maximum projections across 100–200 μm images below the surface through the intact skull, before and after skull optical clearing, showing that the fluorescence intensity is significantly improved. And we could get the information of interneurons after clearing. Scale bar, 100 μm (**b**)**,** 100 μm (**d**), 25 μm (**f**).

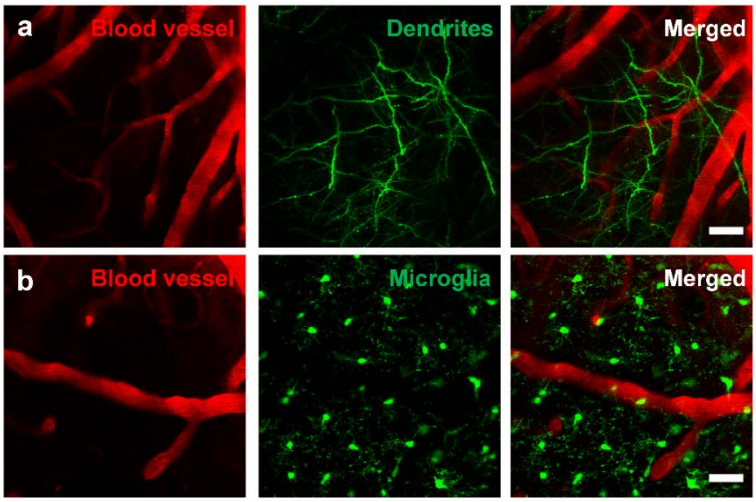

Supplementary Figure 4 Dual-color imaging can be achieved under the SOCW technique. Cerebral vasculature and (**a**) dendrites, (**b**) microglia can be imaged stimultaneously. Scale bar, 25 μm.

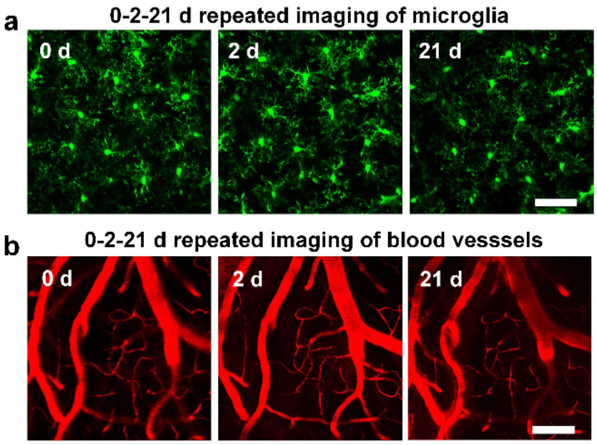

Supplementary Figure 5 Repeated imaging of the microglia and cerebral vasculature through SOCW. (**a**) Repeated imaging of microglia in mice *Cx3cr1*$^{EGFP/+}$ (P28) 0 d, 2d and 21 d after clearing. Scale bar, 50 μm. (**b**) Repeated imaging of brain micro-vessels in *C57BL/6* mice (P28) 0 d, 2d and 21 d after clearing, the plasma had been stained with FITC-dextran. Scale bar, 50 μm.

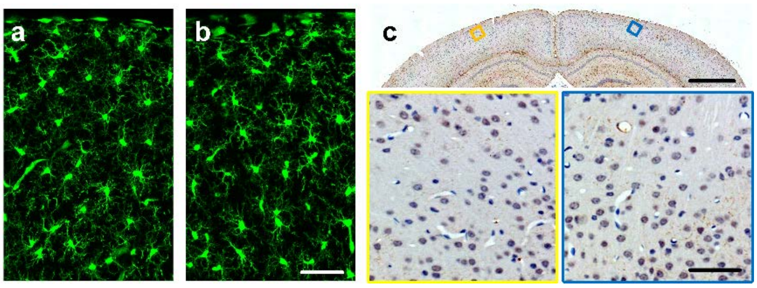

Supplementary Figure 6 Safety assessment of the SOCW technique in adult mice (P60). (**a–b**) Histological images of microglia under SOCW. Two days after clearing, microglia in both the treated (**b**) and control sides (**a**) appear normal. Scale bar, 50 μm. (**c**) Glial fibrillary acidic protein expression under the SOCW technique. The treated and control

hemispheres show similar basal levels of GFAP expression. Scale bar, 1 mm (above), 50 µm (below).

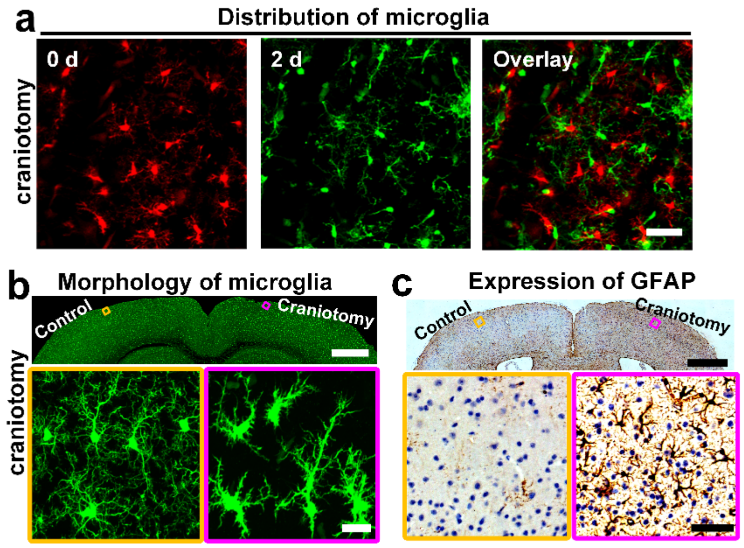

Supplementary Figure 7 Safety assessment of the craniotomy technique. (**a**) Distribution of microglia upon craniotomy. After craniotomy, microglia become active, and their distribution after 2 d is obviously different from that of 0 d. Scale bar, 25 µm. (**b**) Histological images of microglia under craniotomy. Two days after craniotomy, GFP-labeled microglia appeared abnormal, with many ramified branches projected from somata. Scale bar, 1 mm (above), 25 µm (below). (**c**) Ten days after craniotomy, immunostaining showed little GFAP expression in astrocytes on the contralateral control side, but extensive GFAP expression in the entire hemisphere of the cortex subjected to open-skull surgery. Scale bar, 1 mm (above), 50 µm (below).
